# Aquaporin 5 maintains lens transparency by regulating the lysosomal pathway using circRNA

**DOI:** 10.1111/jcmm.17679

**Published:** 2023-02-23

**Authors:** Hu Shaohua, Wang Yihui, Zhang Kaier, Bai Ying, Wang Xiaoyi, Zhao Hui, Di Guohu, Chen Peng

**Affiliations:** ^1^ Department of Human Anatomy, Histology and Embryology, School of Basic Medicine Qingdao University Qingdao China; ^2^ The 971 Hospital of the Chinese People's Liberation Army Navy Qingdao China; ^3^ Institute of Stem Cell Regeneration Medicine, School of Basic Medicine Qingdao University Qingdao China

**Keywords:** aquaporin 5, autophagy, circRNA, HSF4, lens opacity, miRNA, mRNA

## Abstract

The lens is transparent, non‐vascular, elastic and wrapped in a transparent capsule. The lens oppacity of AQP5^−/−^ mice was increased more than that of wild‐type (AQP5^+/+^) mice. In this study, we explored the potential functional role of circular RNAs (circRNAs) and transcription factor HSF4 in lens opacity in aquaporin 5 (AQP5) knockout (AQP5^−/−^) mice. Autophagy was impaired in the lens tissues of AQP5^−/−^ mice. Autophagic lysosomes in lens epithelial cells of AQP5^−/−^ mice were increased compared with AQP5^+/+^ mice, based on analysis by transmission electron microscopy. The genetic information of the mice lens was obtained by high‐throughput sequencing, and then the downstream genes were analysed. A circRNA‐miRNA‐mRNA network related to lysosomal pathway was constructed by the bioinformatics analysis of the differentially expressed circRNAs. Based on the prediction of the TargetScan website and the validation by dual luciferase reporter assay and RNA immunoprecipitation‐qPCR, we found that circRNA (Chr16: 33421321‐33468183+) inhibited the function of HSF4 by sponging microRNA (miR‐149‐5p), and it downregulated the normal expression of lysosome‐related mRNAs. The accumulation of autophagic lysosome may be one of the reasons for the abnormal development of the lens in AQP5^−/−^ mice.

## 1 INTRODUCTION

The lens is the main refractive structure of the eyeball. The transparency of the lens is important.^1^ The inner lens fibre is completely surrounded by the transparent lens capsule. A layer of epithelial cells is attached to the medical surface of the anterior lens capsule. When the epithelial cells reach the equator, they continue to elongate and bend, and become to lens fibres cells.^2^ Degradation of organelles begins in embryonic primary lens epithelial cells (LEC), and it continues in lens fibre cells after birth.^3^, ^4^ The transformation of lens epithelial cells into lens fibre cells occurs throughout life, but its level and speed gradually decrease with age.^4^, ^5^

Aquaporins (AQPs), water‐selective channel proteins, allow water to move rapidly across the plasma membrane. Three aquaporins are expressed in mammalian cornea (*AQP1*, *AQP3* and *AQP5*) and three in the lens (*AQP0*, *AQP1* and *AQP5*).^6^ *AQP5* was expressed in lens epithelial and fibre cells at the gene^7^, ^8^ and protein^9^, ^10^ levels. The subcellular distribution may be orchestrated by its phosphorylation status.^6^ *AQP5* on the cell membrane played an important role in maintaining lens transparency.^11^ In hyperglycaemia caused by diabetes, *AQP5* can maintain lens homeostasis and transparency.^12^ In a previous study, we found that a novel *AQP5* mutation (p. L51P) was related to congenital cataracts, and lens opacity appeared in *AQP5* knockout mice.

Autophagy is an intracellular process that maintains nutritional and energy balance by digesting cytoplasmic components or organelles.^13^ It has been proved that the development of cataract may be related to autophagy, especially macroautophagy.^14^ ATG5 mutations can lead to lens epithelial abnormality and cataract.^15^ Autophagy is a lysosome‐mediated degradation process.^16^, ^17^ Lysosomal hydrolases eventually degrade autophagic substrates. Microtubule‐associated protein 1A/1B‐light chain 3B (LC3B) and a multifunctional scaffolding protein p62/SQSTM1 (p62) are often used to assess autophagy.^18^ Lysosomal components are retrieved to replenish the lysosomal pool after cargo is degraded.^19^ In the lens epithelial and fibre cells, abnormal degradation of organelles caused by dysfunction of lysosomes can cause catract.^20^

In our study, aquaporin 5 knockout (*AQP5* ^−/−^) mice exhibited lens opacity and abnormal autophagy compared with *AQP5* ^+/+^ mice. Differentially expressed circRNAs were screened, and a circRNA‐miRNA‐mRNA network associated with lysosomes was constructed. Our result indicated that *AQP5* deficiency may lead to abnormal lens development, and its mechanism may be related to the disorder of circRNA‐regulated autophagy.

## 2 MATERIALS AND METHODS

### 2.1 Animals and culture of mice primary lens epithelial cells

*AQP5* ^−/−^ (C57BL/6 N) mice were produced through CRISPR/Cas9 technology (Cyagen Biosciences Inc. Guangzhou, China).^21^ All experiments were approved by the Animal Care and Use Committee of Qingdao University (Qingdao, China). Examination with an Ophthalmic slit lamp was performed at 1–12 months of age. Primary lens epithelial cell was prepared from mice aged between 5 and 6 weeks. The lens capsules were separated and digested with 1.5 mg/mL Dispase II enzyme at 37°C for 5 min. After neutralization with complete medium, the cells were centrifuged and suspended and inoculated in Dulbecco's modified Eagle media: Nutrient Mixture F‐12 (Biological Industries) (containing 5 μg/mL TGF‐β, and 2% fetal bovine serum).

### 2.2 Transmission electron microscopy

Lens capsules were isolated and fixed in the electron microscope fixation fluid. They were then fixed with 1% osmium tetroxide. After the samples were dehydrated and embedded, sections of 60–80 nm were made and stained with 2% uranyl acetate solution and lead citrate. The images were collected and analysed under a transmission electron microscope.

### 2.3 Western blot analysis

Dispersion of lens capsules under a microscope was followed by extraction of the proteins with lysis buffer (Beyotime Biotechnology, Shanghai, China). Protein extract was separated by electrophoretic 10% or 12.5% SDS‐PAGE (EPizyme, Shanghai, China). Then, it was transferred to PVDF membranes. The blots were incubated with the primary antibodies of LC3II/I (Abcam, ab192890), P62 (Abclonal, A19700), Lamp1 (Abclonal, A16894), Cln5 (Abclonal, A12886), Gla (Abclonal, A1700) and Lipa (Abclonal, A6385). The blots were incubated with a secondary antibody (ZSGB‐Bio, ZB‐2301), and then they were visualized using enzyme‐linked chemiluminescence using the ECL kit (Applygen, Beijing, China).

### 2.4 Immunofluorescence staining

Immunofluorescence staining was performed on primary LEC and frozen lens sections. They were fixed in 4% paraformaldehyde and then permeated with 0.1% Triton X‐100 (T8200, Solarbio). The samples were stained with primary antibodies and then with secondary antibodies (Thermo Fisher Scientific, Rockford, IL, USA, A11015 and A21207). Nuclei were stained with 4′6‐diamidino‐2‐phenylindole (Beyotime Biotechnology, Shanghai, China).

### 2.5 Extraction and separation of RNA and high‐throughput sequencing

Total RNA was extracted from 6 lenses (combined into one sample, respectively) by using RNA extraction kit (Invitrogen, Carlsbeth, CA, USA). Transcriptome high‐throughput sequencing was performed by Cloud‐Seq Biotech (Shanghai, China). Purified RNA samples were constructed into mice lenses RNA libraries by using the Total RNA Library Preparation Kit (Illumina, San Diego, CA, USA). Then, the 10‐pM library was denatured and reverse‐transcribed into single‐stranded DNA molecules. Finally, the Illumina HiSeq sequencer was used for 150 cycles after setting the parameters.^22^

### 2.6 Analysis of circRNA and mRNA sequencing

HiSeq 4000 sequencer (Illumina) was used to read the paired terminals and then the Q30 was used for quality control. High‐quality reads were mapped using STAR software (version 2.5.1b).^23^ The results were input into the DCC software (version 0.4.4)^24^ and then matched. Unpaired join points were compared with identify candidate circRNAs. Finally, the data were standardized using Edger software (version 3.16.5).^25^ Table 1 showed the preliminary analysis of high‐throughput sequencing results.

**TABLE 1:** Raw reads quality statistical analysis table.

Sample	Raw reads	Q30	Clean reads	Mapped reads	Mapped ratio	CircRNA number
WT1	74592560	0.944645	74503656	47517258	0.637784245	714
WT2	78137096	0.929825	77860806	62510864	0.802854057	1928
WT3	84786612	0.95293	84743764	55359902	0.653262251	1039
KO1	73544066	0.939945	73422408	44400910	0.604732414	593
KO2	69712352	0.946745	69634610	42018526	0.603414394	526
KO3	73966080	0.95067	73878024	46574404	0.630422979	759

### 2.7 Validation of differentially expressed circRNAs, miRNAs and mRNAs

The genes with |log10| > 2 and *p*‐value < 0.05 under the Q30 data set were contained in Table 1. Finally, the miRNAs were predicted by TargetScan (https://www.targetscan.org/) and miRanda (http://www.microrna.org/microrna/), the results were depicted in Tables 2, 3, 4. These together with circRNAs and mRNAs were constructed as the regulation network diagram of circRNA‐miRNA‐mRNA related to the lysosomal pathway. Tables 5 and 6 included primers of circRNAs and mRNAs. Primers for miRNA reverse transcription and qRT‐PCR are listed in Tables 7 and 8. Venn diagram and cluster heat map plot were drawn for circRNAs. Venn diagram, cluster heat map and volcano plot were drawn for mRNAs.

**TABLE 2:** Significantly upregulated and downregulated circRNAs in AQP5^−/−^ mice lens (fold change >2, *p* < 0.05).

circRNAs	CircRNA ID	GeneName	Fold change	Regulation	*p*‐value
1	chr12:102807900‐102812843‐	Btbd7	10.22645799	up	0.022393206
2	chr6:47551732‐47554264‐	Ezh2	10.18790514	up	0.030986708
3	chr11:61744945‐61756677‐	Prpsap2	4.212597297	up	0.040148209
4	chr6:106785257‐106795197‐	Crbn	4.179072104	up	0.040949605
5	chr1:80267050‐80323056‐	Cul3	10.09432134	up	0.046636126
6	chr9:62068385‐62070613‐	Glce	10.09432134	up	0.046636126
7	chr18:78856484‐78859931‐	Setbp1	−10.64445673	down	0.007300008
8	chr10:63287519‐63288009+	Herc4	−10.6042144	down	0.008934374
9	chr7:73514090‐73519708‐	Chd2	−10.53094955	down	0.01386043
10	chr9:7023311‐7050483‐	Dync2h1	−10.49171865	down	0.015599192
11	chr5:23979488‐24000019‐	Fam126a	−10.43235825	down	0.021808935
12	chr8:85875634‐85922242+	Phkb	−10.40914689	down	0.024204907
13	chr7:130196178‐130242644‐	Fgfr2	−10.38968571	down	0.025872905
14	chr4:150439343‐150534945+	Rere	−10.38249825	down	0.027759972
15	chr6:99632257‐99651791‐	Eif4e3	−10.36212377	down	0.030006416
16	chr15:35099434‐35115645‐	Stk3	−10.35333963	down	0.03141662
17	chr7:130418448‐130516168‐	Ate1	−10.33938834	down	0.034443143
18	chr10:63163513‐63169430‐	Mypn	−10.34448535	down	0.032933631
19	chr18:12871078‐12898301‐	Osbpl1a	−10.31431119	down	0.038368551
20	chr16:33421321‐33468183+	Zfp148	−10.28494362	down	0.045408135
21	chr2:73842360‐73846375‐	Atf2	−10.27531973	down	0.047584704
22	chr14:21626719‐21664748+	Kat6b	−11.43246993	down	0.008842788
23	chr5:147450653‐147455188+	Pan3	−11.30944407	down	0.017054586
24	chr3:59031546‐59042413+	Med12l	−10.9524252	down	0.048431958
25	chr2:140042094‐140057499‐	Tasp1	−11.54611576	down	0.020644696
26	chr3:51299783‐51326035‐	Elf2	−11.52464193	down	0.028141323
27	chr6:119920110‐119921028+	Rad52	−11.4769486	down	0.041370034
28	chr5:32478066‐32491189+	Ppp1cb	−11.59400201	down	0.039199648
29	chr2:92214049‐92230724+	Phf21a	−12.18620772	down	0.026228521
30	chr16:33421321‐33434974+	Zfp148	−12.20137172	down	0.039266255

**TABLE 3:** Lysosomal pathway related downregulated expression of mRNAs (fold change > 1.5, *p* < 0.05).

Downregulated mRNAs	Locus	Gene name	Fold change	Regulation	*p*‐value
1	chrX:74297096‐74304721	Atp6ap1	−1.577158878	down	0.00225
2	chr5:124629066‐124725079	Atp6v0a2	−1.501964798	down	0.02795
3	chr8:25944458‐25976744	Hgsnat	−2.177737402	down	0.00125
4	chr12:84754559‐84773112	Npc2	−2.025909881	down	0.00005
5	chr19:34492317‐34527474	Lipa	−3.126459871	down	0.00005
6	chr7:88278084‐88310888	Ctsc	−2.88779834	down	0.00215
7	chr5:129989010‐130003049	Gusb	−3.434571218	down	0.0015
8	chr8:106150398‐106164713	Pla2g15	−2.424807131	down	0.0077
9	chr2:164830731‐164857711	Ctsa	−1.867445319	down	0.0473
10	chr13:97137936‐97198357	Hexb	−1.608566968	down	0.0162
11	chr14:63122461‐63177793	Ctsb	−4.358102113	down	0.00005
12	chr11:55098114‐55113029	Gm2a	−2.056469963	down	0.00005
13	chr13:93770948‐93943016	Arsb	−1.508551553	down	0.03465
14	chr8:85083268‐85098739	Man2b1	−2.079115636	down	0.0004
15	chr7:126571206‐126594941	Cln3	−2.612425689	down	0.0063
16	chr14:103070215‐103077579	Cln5	−1.701925635	down	0.02315
17	chrX:134588148‐134601125	Gla	−2.090923095	down	0.0449
18	chr13:64363213‐64370306	Ctsl	−1.635193084	down	0.00005
19	chr4:117883620‐117887333	Atp6v0b	−1.81416415	down	0.0011
20	chr8:13159134‐13175338	Lamp1	−1.529029244	down	0.0007
21	chr16:10959274‐11066157	Litaf	−1.681610986	down	0.00725
22	chr10:60277626‐60302597	Psap	−2.419116083	down	0.00005
23	chr18:12168716‐12236400	Npc1	−1.877527449	down	0.0168
24	chr6:122308719‐122317680	M6pr	−1.842633312	down	0.0006
25	chr10:121365089‐121397253	Gns	−1.592507316	down	0.0022
26	chr11:4986823‐5042791	Ap1b1	−1.568382011	down	0.01455
27	chr7:142325836‐142388038	Ctsd	−1.794639412	down	0.00005
28	chr7:81460398‐81493925	Ap3b2	−1.681468789	down	0.02315
29	chr1:5083172‐5162549	Atp6v1h	−1.844089914	down	0.008

**TABLE 4:** Top 5 miRNAs for each circRNA.

circRNA	miRNA	miRNA	miRNA	miRNA	miRNA
chr5:147450653‐147455188+	mmu‐miR‐6992‐5p	mmu‐miR‐320‐3p	mmu‐miR‐8104	mmu‐miR‐1950	mmu‐miR‐3473f
chr11:61744945‐61756677‐	mmu‐miR‐7094‐1‐5p	mmu‐miR‐337‐3p	mmu‐miR‐499‐3p	mmu‐miR‐7054‐5p	mmu‐miR‐7116‐3p
chr12:102807900‐102812843‐	mmu‐miR‐130a‐5p	mmu‐miR‐6957‐3p	mmu‐miR‐6946‐3p	mmu‐let‐7 e‐5p	mmu‐miR‐342‐3p
chr2:73842360‐73846375‐	mmu‐miR‐7054‐5p	mmu‐miR‐5616‐3p	mmu‐miR‐7012‐5p	mmu‐miR‐672‐5p	mmu‐miR‐134‐5p
chr5:32478066‐32491189+	mmu‐miR‐29b‐2‐5p	mmu‐miR‐6338	mmu‐miR‐7065‐3p	mmu‐miR‐7a‐1‐3p	mmu‐miR‐7013‐3p
chr18:12871078‐12898301‐	mmu‐miR‐6898‐3p	mmu‐miR‐201‐3p	mmu‐miR‐26a‐2‐3p	mmu‐miR‐683	mmu‐miR‐7067‐3p
chr2:140042094‐140057499‐	mmu‐miR‐207	mmu‐miR‐1907	mmu‐miR‐497a‐5p	mmu‐miR‐1906	mmu‐miR‐15a‐5p
chr1:80267050‐80323056‐	mmu‐miR‐6981‐5p	mmu‐miR‐466i‐3p	mmu‐miR‐466o‐3p	mmu‐miR‐7661‐5p	mmu‐miR‐5110
chr4:150439343‐150534945+	mmu‐miR‐5110	mmu‐miR‐6353	mmu‐miR‐27b‐3p	mmu‐miR‐322‐5p	mmu‐miR‐3085‐5p
chr6:106785257‐106795197‐	mmu‐miR‐466i‐5p	mmu‐miR‐466c‐5p	mmu‐miR‐466o‐5p	mmu‐miR‐466b‐5p	mmu‐miR‐7226‐5p
chr16:33421321‐33434974+	mmu‐miR‐17‐3p	mmu‐miR‐6925‐5p	mmu‐miR‐106a‐3p	mmu‐let‐7j	mmu‐miR‐20b‐3p
chr6:119920110‐119921028+	mmu‐miR‐152‐5p	mmu‐miR‐3084‐5p	mmu‐miR‐188‐3p	mmu‐miR‐7068‐5p	mmu‐miR‐1904
chr6:47551732‐47554264‐	mmu‐miR‐6946‐3p	mmu‐miR‐6938‐3p	mmu‐miR‐7210‐5p	mmu‐miR‐6935‐3p	mmu‐miR‐598‐3p
chr3:51299783‐51326035‐	mmu‐miR‐149‐5p	mmu‐miR‐7039‐3p	mmu‐miR‐7684‐5p	mmu‐miR‐7676‐5p	mmu‐miR‐6932‐3p
chr5:23979488‐24000019‐	mmu‐miR‐7019‐5p	mmu‐miR‐6958‐3p	mmu‐miR‐767	mmu‐miR‐7021‐3p	mmu‐miR‐8113
chr2:92214049‐92230724+	mmu‐miR‐432	mmu‐miR‐6971‐3p	mmu‐miR‐448‐3p	mmu‐miR‐337‐3p	mmu‐miR‐16‐1‐3p
chr15:35099434‐35115645‐	mmu‐miR‐669a‐3p	mmu‐miR‐669o‐3p	mmu‐miR‐6897‐3p	mmu‐miR‐137‐3p	mmu‐miR‐207
chr10:63287519‐63288009+	mmu‐miR‐3970	mmu‐miR‐3472	mmu‐miR‐125a‐5p	mmu‐miR‐875‐3p	mmu‐miR‐26a‐2‐3p
chr6:99632257‐99651791‐	mmu‐miR‐7032‐3p	mmu‐miR‐6975‐3p	mmu‐miR‐7013‐3p	mmu‐miR‐26a‐2‐3p	mmu‐miR‐6940‐5p
chr7:73514090‐73519708‐	mmu‐miR‐6963‐3p	mmu‐miR‐6908‐3p	mmu‐miR‐7011‐3p	mmu‐miR‐8106	mmu‐miR‐7231‐3p
chr14:21626719‐21664748+	mmu‐miR‐320‐5p	mmu‐miR‐6961‐3p	mmu‐miR‐1903	mmu‐miR‐877‐3p	mmu‐miR‐7009‐3p
chr3:59031546‐59042413+	mmu‐miR‐3064‐5p	mmu‐miR‐6924‐5p	mmu‐miR‐6969‐5p	mmu‐miR‐367‐3p	mmu‐miR‐93‐3p
chr18:78856484‐78859931‐	mmu‐miR‐3473b	mmu‐miR‐7669‐3p	mmu‐miR‐3473 e	mmu‐miR‐6940‐5p	mmu‐miR‐6914‐5p
chr18:78856484‐78859931‐	mmu‐miR‐17‐3p	mmu‐miR‐7660‐3p	mmu‐miR‐7235‐3p	mmu‐miR‐1896	mmu‐miR‐149‐5p
chr9:62068385‐62070613‐	mmu‐miR‐291b‐5p	mmu‐miR‐291a‐5p	mmu‐miR‐7015‐5p	mmu‐miR‐6930‐5p	mmu‐miR‐6950‐3p
chr9:62068385‐62070613‐	mmu‐miR‐880‐3p	mmu‐miR‐344d‐2‐5p	mmu‐miR‐1946b	mmu‐miR‐7115‐3p	mmu‐miR‐680
chr9:7023311‐7050483‐	mmu‐miR‐6964‐3p	mmu‐miR‐7234‐5p	mmu‐miR‐26a‐2‐3p	mmu‐miR‐30d‐3p	mmu‐miR‐7680‐3p
chr10:63163513‐63169430‐	mmu‐miR‐298‐5p	mmu‐miR‐877‐3p	mmu‐miR‐6915‐5p	mmu‐miR‐3083‐5p	mmu‐miR‐7053‐3p
chr7:130418448‐130516168‐	mmu‐miR‐6984‐3p	mmu‐miR‐141‐5p	mmu‐miR‐7116‐3p	mmu‐miR‐6946‐3p	mmu‐miR‐1903

**TABLE 5:** Primers used in circRNA.

Gene	Primer type	Primer sequence
chr4: 150439343‐150534945+	Forward	GCCCAAGCTGATCGAGAAGT
	Reverse	CACAGAAAGCAGGAGCTGGA
chr18: 12871078‐12898301‐	Forward	AGAGACTTGTGTGGCGTTGA
	Reverse	TCGAATGGAGAAGAGGCAGC
chr3: 59031546‐59042413+	Forward	TTAAGAAACGCCAGGCTCCC
	Reverse	GCCATGTTCGTCTCCAGTGA
chr2: 140042094‐140057499‐	Forward	AACGAGCTTGTCAGAAGGCA
	Reverse	CCAACTGTGCAAAGGGAGTC
chr6: 119920110‐119921028+	Forward	AAGAAAAGCCTGGACCTGGG
	Reverse	CAGTTACAGGGGTGCTGTGT
GAPDH	Forward	AAGGTCATCCCAGAGCTGAA
	Reverse	CTGCTTCACCACCTTCTTGA

**TABLE 6:** Primers used in mRNA.

Gene	Primer type	Primer sequence
Lipa	Forward	TCCAGGATCTGCCTGTCTCT
	Reverse	GGCTTGCTACACAAGCATGA
Gm2a	Forward	GCAGGCCTCTTCTTCTGTGT
	Reverse	TCCATCCTCCCTCAGGTCAG
Gla	Forward	TGCCTGCATAAGTGAGCAAC
	Reverse	GTGGACGTAATTTGCGAGGT
Lamp1	Forward	TCTTCAGTGTGCAGGTCCAG
	Reverse	ATGAGGACGATGAGGACCAG
Hgsnat	Forward	GTTCTTCTGCGAACCGTCTC
	Reverse	ACAAACCATGGGAAGACGAG
Cln3	Forward	GCCTTCACTTGCTGCCTTAC
	Reverse	CACACAGGCTTAACCCCACT
Cln5	Forward	AGCTGGCTGCTTCTTGAGAC
	Reverse	AGCTGGCTGCTTCTTGAGAC
Npc2	Forward	GCCAATACTTGGTTGCAGGT
	Reverse	ACTGAACACACACGCAGGAG
Ctsb	Forward	TGCATCTTGAAGCTGGTCAC
	Reverse	GAGCTGGCCACATTACCAAT
litaf	Forward	CTGGGAACTCCCTTTGTTCA
	Reverse	GGATCCCCTAGGAGTTCGAG
HSF4	Forward	GTACAACGTCACCGAGAGCA
	Reverse	GCTTTTTCAGAGGGATGCAG
GAPDH	Forward	AAGGTCATCCCAGAGCTGAA
	Reverse	CTGCTTCACCACCTTCTTGA

**TABLE 7:** RT primers of miRNA used in reverse transcription.

Gene	Primer sequence
miR‐149‐5p	GTCGTATCCAGTGCGTGTCGTGGAGTCGGCAATTGCACTGGATACGACAAAGTC
U6	AACGCTTCACGAATTTGCGT

**TABLE 8:** Primers of miRNA used in qRT‐PCR.

Gene	Primer type	Primer sequence
miR‐149‐5p	Forward	GGGTCTGGCTCCGTGTCTTC
	Reverse	CAGTGCGTGTCGTGGAGT
U6	Forward	CTCGCTTCGGCAGCACA
	Reverse	AACGCTTCACGAATTTGCGT

### 2.8 Gene Ontology (GO) and the Kyoto Encyclopedia of Genes and Genomes (KEGG) pathway analysis

High quality reads were uploaded to STAR software (version 2.5.1b) (http://www.bioinfo‐scrounger.com/). Through GO (http://www.geneontology.org) analysis the function of mRNA was analysed from three aspects: molecular function, biological processes and cell composition. KEGG (http://www.genome.jp/kegg) pathway enrichment analysis was used to explain the pathways of the differentially expressed genes. *p* < 0.05 was considered significant. The top 10 enhanced GO terms were ranked according to their *p*‐values.

### 2.9 RNA immunoprecipitation‐qPCR

The expression difference of Chr16: 33421321‐33468183+, mmu‐miR‐149‐5p, and HSF4 were analysed using the EZ‐Magna RIP RNA‐Binding Protein Immunoprecipitation kit (Millipore, Bedford, MA United States). The lens was lysed into cell suspensions in RIP lysis buffer. The extract was then incubated with immunoprecipitation buffer containing magnetic beads conjugated with Anti‐Ago2 (ab186733, Abcam, Cambridge, MA, USA) or Anti‐IgG (ab181236, Abcam). The beads were collected and washed. The RNA complex was isolated by phenol–chloroform extraction. The enrichment levels of target genes were analysed by qRT‐PCR. Tables 5 and 6 show the primers used for selected circRNAs and mRNAs.

### 2.10 A dual luciferase reporter assay

The TargetScan database was employed to predict the possible binding sites of HSF4 and mmu‐miR‐149‐5p which might target the 3′UTR of HSF4 at three positions: 159–165, 500–506 and 517–523. To determine the interaction between mmu‐miR‐149‐5p and HSF4, the wild‐type and mutated target sequence of HSF4 were cloned into luciferase vector plasmids (Genome Editech, Shanghai, China). The constructs were wild‐type HSF4 (HSF4 WT) and HSF4 mutant 1 (159–165) (HSF4 MT1), HSF4 mutant 2 (500–506) (HSF4 MT2) and HSF4 mutant 3 (517–523) (HSF4 MT3). For the luciferase assay, HEK‐293 cells were co‐transfected with (1) HSF4 WT together with the NC mimics or miR‐149‐5p mimics; (2) HSF4 MT1 together with the NC mimics or miR‐149‐5p mimics; (3) HSF4 MT2 together with the NC mimics or miR‐149‐5p mimics; (4) HSF4 MT3 together with the NC mimics or miR‐149‐5p mimics. Lipofectamine 2000 (Invitrogen) was used to perform the transfection according to the manufacture's protocol. According to the instructions and prior to standardization of Renilla luciferase internal control, fireflies and Renilla luciferase activity were analysed using a dual luciferase reporting kit (Promega, Madison, WI, USA).^11^

### 2.11 Statistical analysis

Statistical analysis was performed using GraphPad Prism 8.0 (GraphPad Software Inc., La Jolla, CA, United States). The results were presented as mean ± SD. Comparison between two groups was assessed using Student's *t*‐test (*p* < 0.05 considered significant). All experiments were repeated three times.

## 3 RESULTS

### 3.1 Lens opacity and expression of circRNA in lenses

**FIGURE 1:**
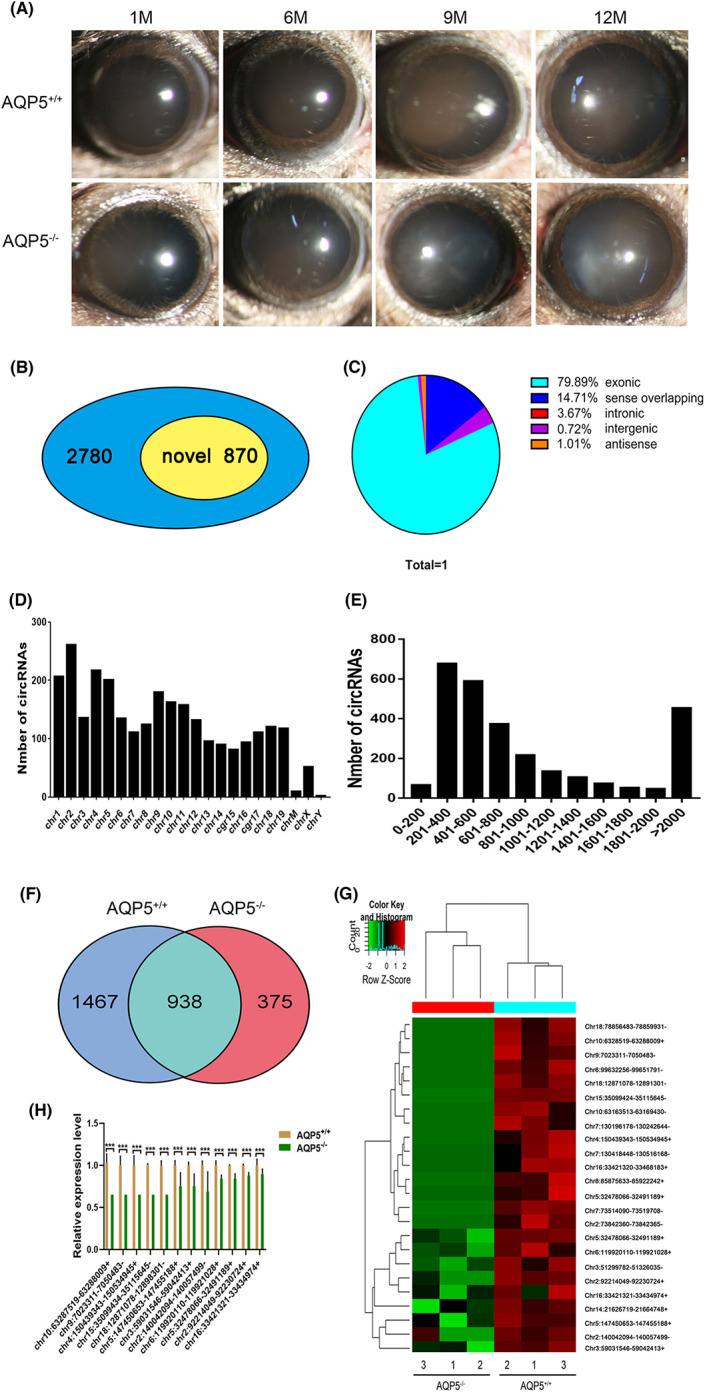
Expression pattern of circRNAs and differential expressed circRNA. (A) Effect of *AQP5* on the lens transparency. Slit‐lamp images of the eyes from *AQP5* ^+/+^ and age‐matched *AQP5* ^−/−^ mice (1 M, 6 M, 9 M and 12 M); (B) The proportion of newly discovered circRNAs in all identified circRNAs; (C) The genomic origin of detected circRNAs; (D) Distribution of circRNAs on chromosomes; (E) The length distribution of exonic circRNAs; (F) Venn diagram of differentially expressed circRNAs; (G) the profile of circRNAs expression analysed by ierarchical clustering in *AQP5* ^−/−^ mice (*n* = 3) versus *AQP5* ^+/+^ mice (*n* = 3). (H) The expression of twelve downregulated circRNAs was detected by quantitative real‐time polymerase chain reaction in *AQP5* ^−/−^ mice. *p < 0.05, **p < 0.01, ***p < 0.001.

Through observation under slit lamp, it was found that the lens opacity of *AQP5* ^−/−^ mice increased with age (Figure 1A). To investigate the possible mechanism of AQP5 deficiency on lens transparency, RNA was obtained from the lens of mice and sequencing was performed. A total of 2780 circRNAs were present in the lenses, of which 870 had not been reported before (Figure 1B). There were many types of circRNAs, with exons accounting for about 80% of the total circRNAs (Figure 1C). Majority of the circRNAs were on chromosomes 1–19 (Figure 1D). The size of circRNAs varied greatly, ranging from 128 nucleotides to more than 2000 nucleotides (Figure 1E). The total mean length was 3453 nt. Of the 2780 circRNAs identified; 1476 were detected only in *AQP5* ^*+/+*^ mice and 375 were checked only in *AQP5* ^*−/−*^ mice (Figure 1F). 30 differentially expressed circRNAs were found in *AQP5* ^−/−^ mice, of which 24 were downregulated and 6 were upregulated. Hierarchical clustering indicated significant differences in the expressions of circRNAs between *AQP5* ^+/+^ and *AQP5* ^−/−^ mice (Figure 1G). The downregulation of 12 circRNAs was verified by Quantitative real‐time polymerase chain reaction (Figure 1H).

### 3.2 Functional prediction of differentially expressed circRNAs in the lenses

**FIGURE 2:**
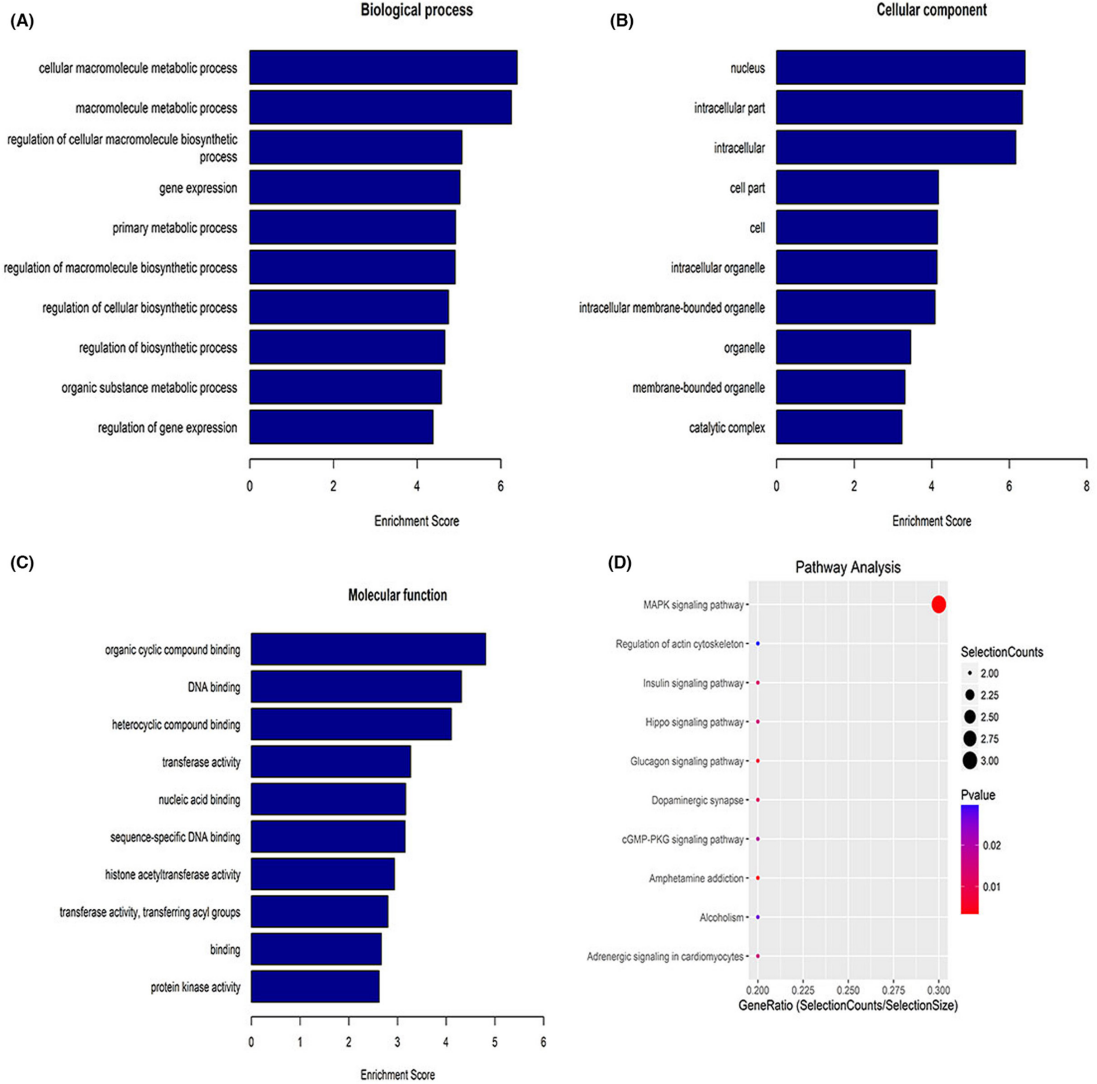
GO analysis and KEGG pathway analysis of downregulated circRNA. (A) Biological processes; (B) cellular components; (C) molecular functions; (D) relevant pathways were identified for downregulated circRNAs.

We performed pathway enrichment analyses using GO and the KEGG. Cellular macromolecule metabolic process was the most abundant GO terms for downregulated circRNAs (Figure 2A), nucleus (Figure 2B) and organic cyclic compound binding (Figure 2C). The most relevant pathway was the MAPK signalling pathway (Figure 2D).

### 3.3 Differentially expressed mRNAs in the lens

**FIGURE 3:**
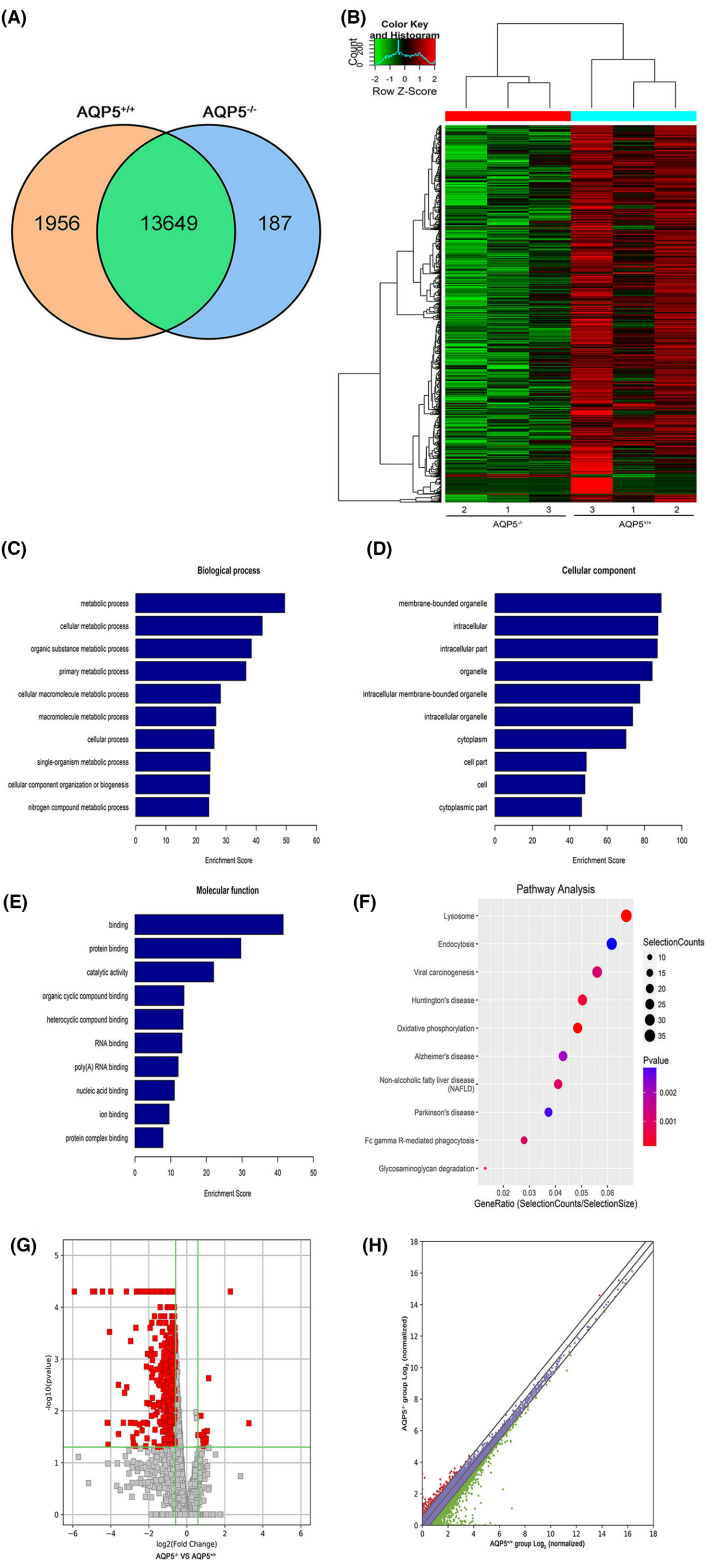
GO analysis and KEGG pathway analysis of downregulated mRNA in the lens of *AQP5* ^−/−^ mice. (A) Venn diagrams of total mRNA in *AQP5* ^+/+^ and *AQP5* ^−/−^ mice. (B) Hierarchical clustering displayed the mRNA expression profile of the *AQP5* ^−/−^ mice (*n* = 3) versus the *AQP5* ^+/+^ mice (*n* = 3); (C) biological processes; (D) cellular components; (E) molecular functions; (F) relevant pathways were identified for downregulated mRNA. (G) volcano plot of differentially expressed mRNAs. (H) Scatter plot of differentially expressed mRNAs.

A total of 15,792 mRNAs were found in the mouse lenses, of which 1956 were expressed in *AQP5* ^+/+^ mice, and 187 were expressed in *AQP5* ^−/−^ mice (Figure 3A). 1214 differentially expressed mRNAs were screened from *AQP5* ^−/−^ mice, of which 14 were significantly upregulated and 1200 were remarkably downregulated more than 2‐fold. Hierarchical cluster analysis showed that the mRNA expression profiles of the lens of both types of mice were significantly different (Figure 3B). The most abundant GO term for downregulated mRNAs were in response to metabolic processes (Figure 3C), membrane‐bounded organelle (Figure 3D) and binding (Figure 3E). KEGG analysis showed that the most relevant pathway of downregulated mRNAs was the lysosome (|log_2_ FC| ≥ 1 and *p*‐value < 0.05) (Figure 3F). The volcano plot showed that the differential expression of mRNAs in *AQP5* ^+/+^and *AQP5* ^−/−^ mice was significant (|log_2_ FC| ≥ 1 and *p*‐value < 0.05) (Figure 3G). The scatter plot was built to assess the expression variation of mRNAs between the two groups (|log_2_ FC| ≥ 1 and *p*‐value < 0.05) (Figure 3H).

### 3.4 Analysis of the circRNA‐miRNA‐mRNA network

**FIGURE 4:**
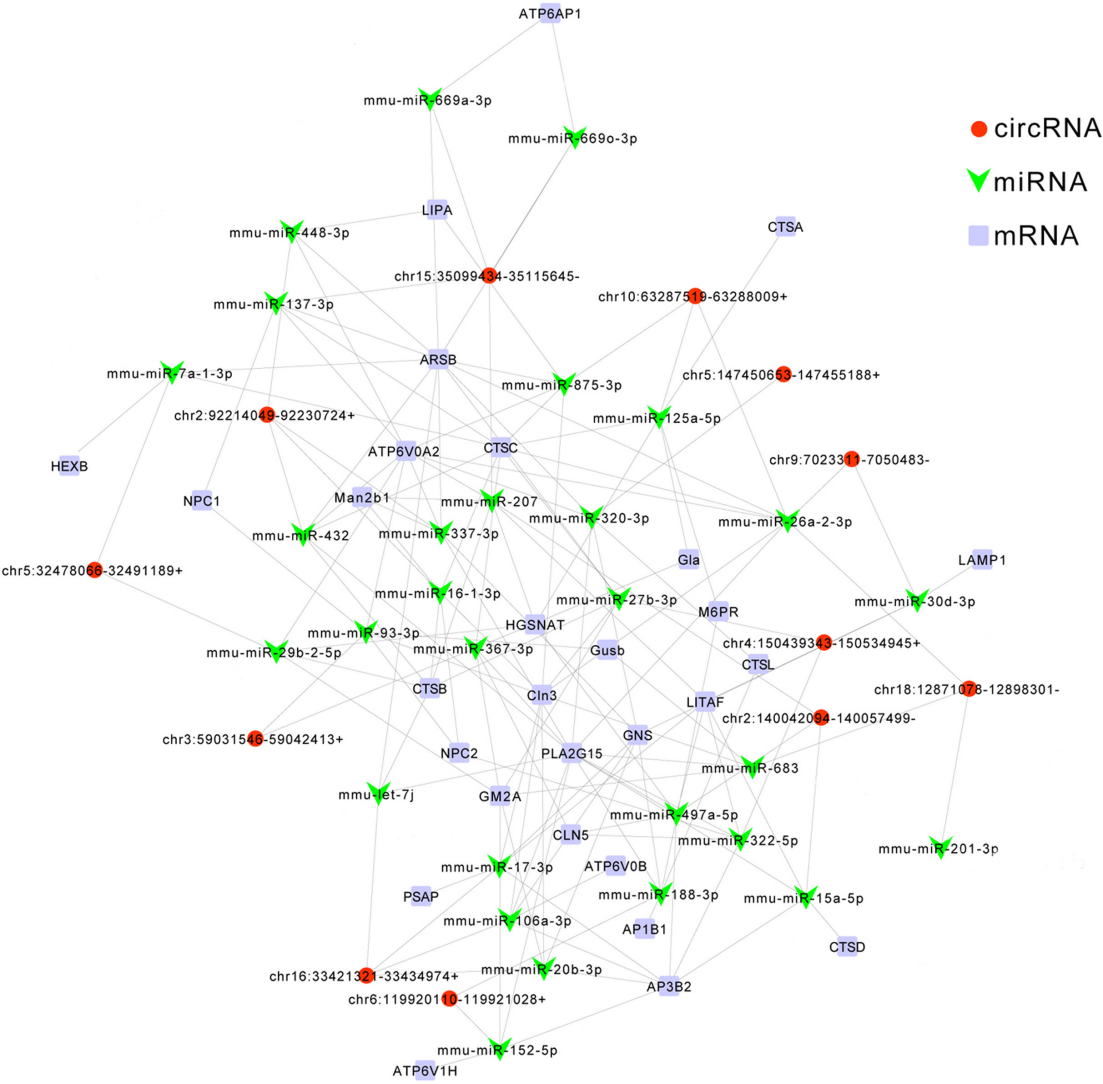
circRNA‐miRNA‐mRNA network. Red circles represented circRNAs, green arrowhead represented miRNAs and purple squares represented mRNAs.

A total of 12 circRNAs with reduced expression were selected as predictive miRNA binding sites. The method of mRNA selection was the same as that of circRNA screening, and 29 lysosomal associated mRNAs with decreased expression in microarray results were screened in the *AQP5* ^−/−^ group. The 29 miRNAs were identified by TargetScan and miRanda. All Target circRNAs, miRNAs and mRNAs were selected, and then the network relationship diagram between circRNA‐miRNA and miRNA‐mRNA association pairs was constructed (Figure 4).

### 3.5 Validation of the lysosomal pathway and abnormal autophagy in *AQP5* ^−/−^ mice

**FIGURE 5:**
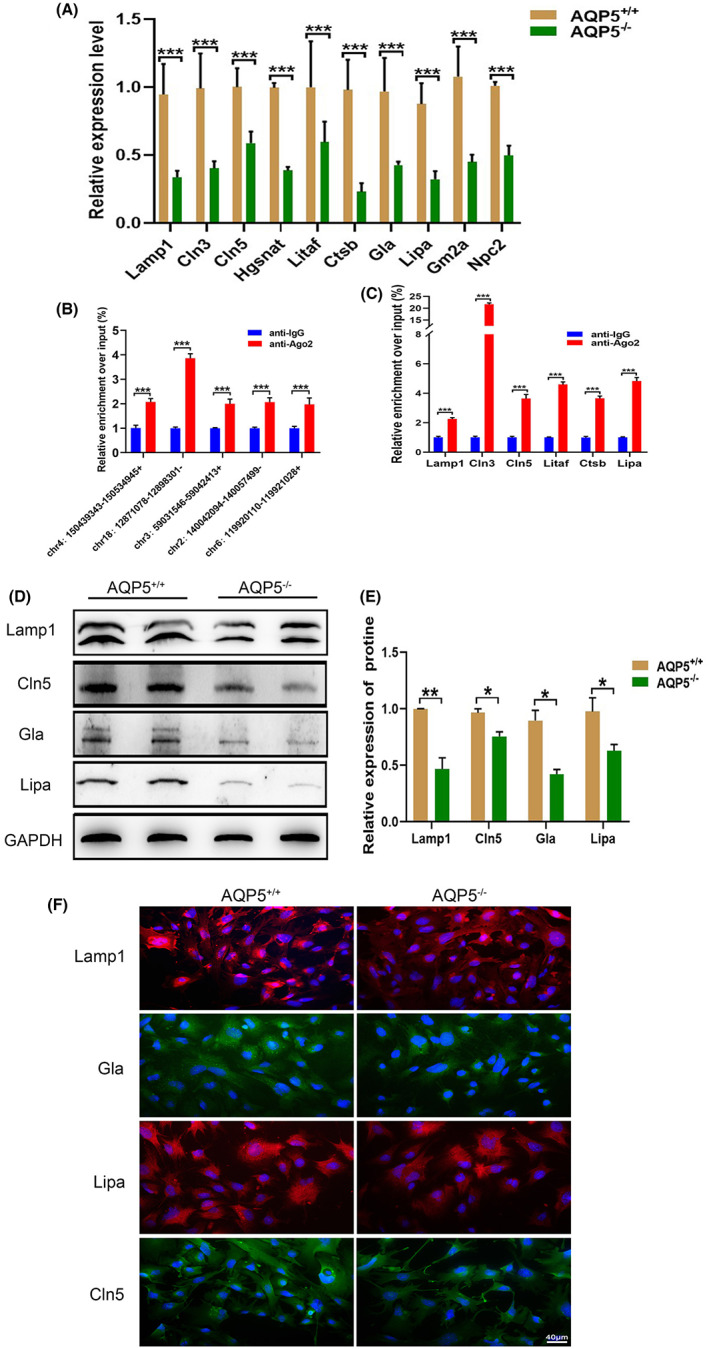
Validation of the lysosomal pathway. (A) 10 downregulated mRNAs in the lens of *AQP5* ^−/−^ was detected by qRT‐PCR. (B) RIP‐qPCR analysis of five cirRNAs Chr4:150439343‐150534945+; Chr18:12871078‐12898301‐; Chr3:59031546‐59042413+; Chr2:140042094‐140057499‐; Chr6:119920110‐119921028+; (C) RIP‐qPCR analysis of six mRNAs Lamp1, Cln3, Cln5, Litaf, Ctsb, Lipa. anti‐IgG, immunoglobulin G negative control group. (D) Western blot bands of Lamp1, Cln5, Gla, Lipa; (E) Quantified intensities of Western blot bands of Lamp1, Cln5, Gla, Lipa (*N* = 3 samples). (F) Immunofluorescence staining showed the expression of Lamp1, Cln5, Gla and Lipa in the primary lens epithelial cells (red: Lamp1, Lipa; green: Gla, Cln5; blue: DAPI). Scale bars: (F) 40 μm. *p < 0.05, **p < 0.01, ***p < 0.001.

A qRT‐PCR experiment was performed on 10 mRNAs of the lysosomal pathway (membrane proteins: Lamp1, Cln3, Cln5, Hgsnat and Lipaf; enzymes: Ctsb, Gla, Lipa, Gm2a, and Npc2). It showed that the relative expression levels of the 10 mRNAs were downregulated in *AQP5* ^−/−^ group (Figure 5A). The RIP‐qPCR experiment showed that the enrichment of Chr4:150439343‐150534945+, Chr18:12871078‐12898301‐, Chr3: 59031546‐59042413+, Chr2:140042094‐140057499‐ and Chr6: 119920110‐119921028+ were elevated in the anti‐Ago2 group (Figure 5B). RIP‐qPCR experiment also showed that the enrichment of Lamp1, Cln3, Cln5, Litaf, Ctsb and Lipa was elevated in the anti‐Ago2 group (*p* < 0.05) (Figure 5C). The expression of lysosomal proteins Lamp1, Cln5, Gla and Lipa were less in the lenses of *AQP5* ^−/−^ mice than that of AQP5^+/+^ mice (Figure 5D–F).

**FIGURE 6:**
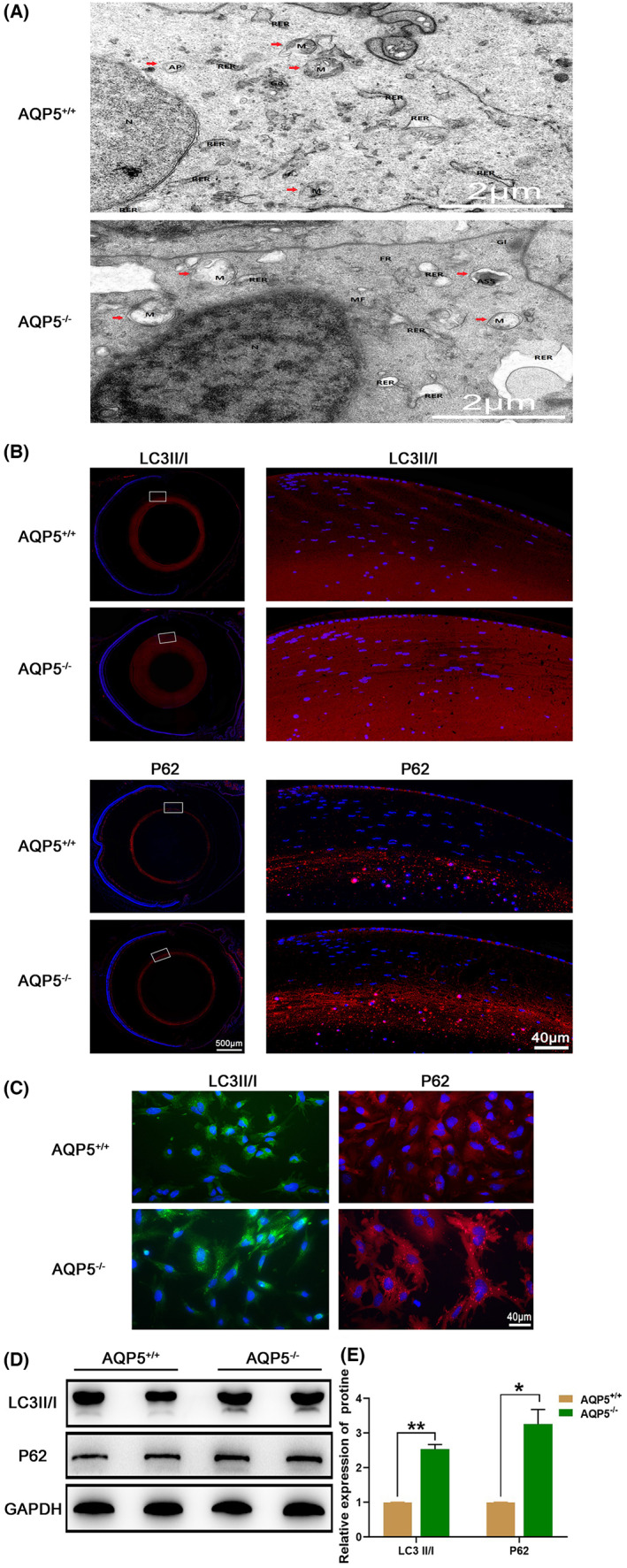
Effect of *AQP5* on the lens autophagy. (A) Ultrastructure in the lens epithelial cells under transmission electron microscopy (TEM). nucleus (N), autophagic lysosomes (ASS), mitochondria (M) and rough endoplasmic reticulum (RER). Scale bars: 2 μm; (B.C) Immunofluorescence staining of LC3II/I in the lens (red: LC3II/I, blue: DAPI), and in the mice primary lens epithelial cell location (green: LC3II/I, blue: DAPI); Immunofluorescence staining of P62 in the lens and in the mice primary lens epithelial cells location (red: P62, blue: DAPI); Scale bars: 500 μm, 60 μm, 40 μm. *p < 0.05, **p < 0.01, ***p < 0.001. (D) Abnormal autophagy in lens of *AQP5* ^−/−^ mice. Western blot bands for LC3II/I, P62 and GAPDH; (E) Quantified intensities of Western blot bands for LC3II/I, P62 compared with GAPDH (*N* = 3 samples).

Transmission electron microscopy showed that the structure of organelles in LEC of *AQP5* ^+/+^ mice were generally normal. However, an autophagic lysosome (ASS) was found in *AQP5* ^−/−^ mice (Figure 6A). The results of immunofluorescence staining showed that LC3II/I and p62 were widely expressed in mouse lens epithelial and fibre cells (Figure 6B). In addition, the fluorescence of LC3II/I and p62 in the primary cultured lens epithelial cells of *AQP5* ^−/−^ mice seemed to be brighter than that of *AQP5* ^*+/+*^ mice (Figure 6C). To quantitatively compare the expression levels of LC3II/I and p62, we performed a Western blot assay. LC3II/I and P62 were greatly increased in the lenses of *AQP5* ^−/−^ mice compared with the *AQP5* ^*+/+*^ mice (Figure 6D,E).

### 3.6 Transcription factor HSF4 participated in the regulation of lysosomal pathway mRNAs

**FIGURE 7:**
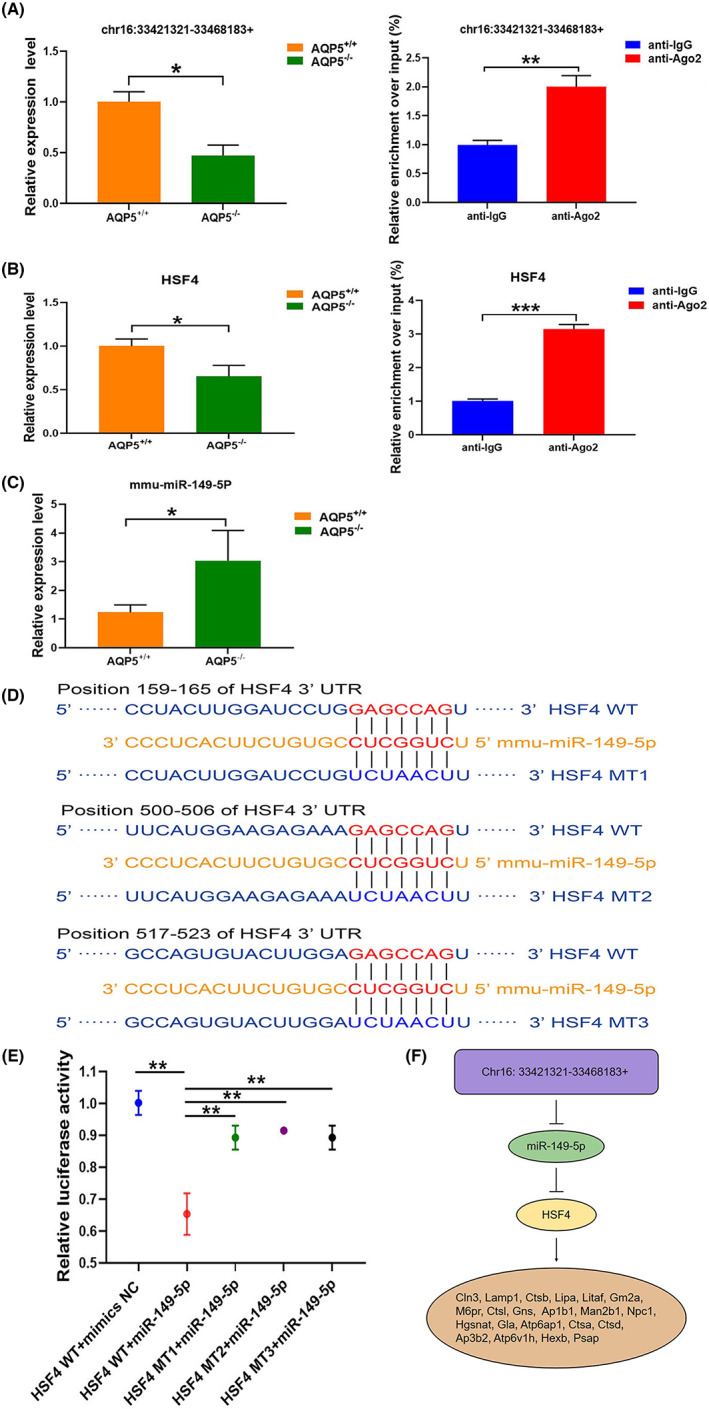
HSF4 was involved in the regulation of lysosome pathway mRNA. (A) Chr16: 33421321‐33468183+ was detected by qRT‐PCR and RIP‐qPCR; (B) HSF4 was detected by qRT‐PCR and RIP‐qPCR; (C) miR‐149‐5p was detected by qRT‐PCR; (D) position of sequence targeted by miR‐149‐5p in the 3′‐UTR of HSF4; (E) a dual luciferase activity reporter assay for the verification of HSF4 as a direct target gene of miR‐149‐5p. Data were shown as mean ± SD (*N* = 3 per group). Mt, mutant; NC, negative control; WT, wild type. (F) A regulatory pattern diagram of HSF4 in the *AQP5* ^−/−^ lens. *p < 0.05, **p < 0.01, ***p < 0.001.

QRT‐PCR experiment showed that Chr16: 33421321‐33468183+ was decreased in *AQP5* ^−/−^ mice (*p* < 0.05). RIP‐qPCR experiment showed that the enrichment of chr16:33421321‐33468183+ was elevated in the anti‐Ago2 group (*p* < 0.05) (Figure 7A). qRT‐PCR experiment also showed that HSF4 expression was decreased in *AQP5* ^−/−^ mice (*p* < 0.05). RIP‐qPCR experiment showed that enrichment of HSF4 was elevated in the anti‐Ago2 group (*p* < 0.05) (Figure 7B). QRT‐PCR experiment showed that miR‐149‐5p was increased in the lens of *AQP5* ^−/−^ mice (*p* < 0.05) (Figure 7C). With the aim of further exploring the mitigating effect of the downstream regulatory mechanism of HSF4 on lens opacity, the upstream miRNAs of HSF4 were screened by the TargetScan database. It revealed that miR‐149‐5p had better prediction results. Therefore, miR‐149‐5p was selected for further studies. Three mutation sites were designed according to the predicted three binding sites (Figure 7D). A luciferase reporter assay showed that, in the presence of HSF4‐WT‐UTR and miR‐149‐5p mimics, the relative luciferase activity was significantly antagonized (Figure 7E). Mutations of the miR‐149‐5p complementary sites in the 3′UTR of HSF4 (HSF4M1, HSF4M3, HSF4M3) abolished the suppressive effect of miR‐149‐5p through the disruption of the interaction between miR‐149‐5p and HSF4 (Figure 7E). The analysis showed that a regulatory pattern diagram of HSF4 was derived. Chr16: 33421321‐33468183+ probably adsorbed to miR‐149‐5p through the sponge mechanism, and miR‐149‐5p unidirectional regulate HSF4 expression finally targeting regulation of lysosomal mRNAs (Figure 7F).

## 4 DISCUSSION

Thirteen human AQP subtypes have been identified. Current studies have shown that the functional differences in water permeability in different tissues and cells may be related to transcriptional regulation, post‐translational modification, protein stability and polarized membrane distribution among different AQP subtypes.^26^ AQP0, AQP1, and AQP5 are conserved in all mammalian lens examined thus far.^7^, ^27^

Seven AQP0 mutations that can cause cataract have been found, all of which lead to the inability of AQP0 to traffic to the plasma membrane.^28^, ^29^ These mutations include Arg233Lys, Arg33Cys, Asp150His, Glu134Gly, Thr138Arg, as well as C‐terminal truncation mutants, Δ213‐AQP0 and Tyr219stop.^30^ The expression of AQP1 increased gradually after birth, which was consistent with the increase in lens size during growth and development.^31^ The water permeability of lens epithelium in AQP1^−/−^ mice was approximately three times lower.^32^ AQP5 is expressed in cornea, lacrimal gland, lens, lung pneumocyte type I cells, retina, salivary gland, pancreas and uterus.^33^ AQP5 is less abundant, but the water permeability of AQP5 is 20 times that of AQP0.^34^ We previously found that the *AQP5* ^−/−^ mice developed lens opacity, which increased with age. In this study, we found that the number of autophagic lysosome in the lens epithelial cells of *AQP5* ^−/−^ mice was more than the *AQP5* ^+/+^ mice.

As development proceeds, the differentiation of fibre cells is accompanied by degrading membranous organelles such as nuclei, mitochondria, Golgi apparatus and endoplasmic reticulum,^35^ form organelle‐free zones, namely organelle‐free zones^36^ to achieve optical transmittance.^37^ Autophagosomes are elucidated from immunoelectron microscopy findings in lens epithelial cells.^38^ Later studies revealed autophagosomes existed in lens epithelial and fibre cells of mice, chicken and humans.^39^ P62 serves as a scaffold having binding sites for both ubiquitin and LC3B.^40^ LC3B and p62 are degraded by hydrolysis enzymes of lysosomes along with the cargo.^41^, ^42^, ^43^ The correlation between autophagy and lens development and cataract formation has been reported, but there are few studies on the relationship between AQP5 and autophagy in lens development. In the study, we found that extended outflow blockage led to extensive accumulation of p62 protein in the lens of AQP5^−/−^ mice (Figure 6B–E), while the expression of Lamp1, Lipa, Gla and Cln5 decreased than those of *AQP5* ^+/+^ mice through Western blot and immunofluorescence staining (Figure 5D–F). The same result was found in the primary LEC.

There is no evidence of how AQP5 causes the change of autophagy level. The relationship between AQP5 and autophagy needs to be clarified. CircRNAs, a novel class of noncoding RNAs,^44^, ^45^ can regulate target genes through miRNA sponge or RNA‐binding proteins.^46^, ^47^ CircRNAs participates in pathological process such as apoptosis, proliferation, activity and oxidative damage and may eventually cause cataract.^48^ Experimental studies have shown that they could be specifically several miRNAs, like miR‐15a, miR‐23b‐3p, miR‐34a‐5p, miR‐184 and miR‐211‐5p and regulate the apoptosis or oxidative stress of lens epithelial cells in the formation of catract.^49^, ^50^ The regulation of target mRNAs by miRNA is usually achieved by guiding the degradation or inhibiting their translation.^51^

More and more evidence show that circRNAs are involved in the occurrence, pathogenesis and progression of various ocular disorders.^52^ In our study, we found 12 downregulated circRNAs, 29 upregulated miRNAs and 29 downregulated mRNAs constructed a circRNA‐miRNA‐mRNA regulatory network. RIP‐qPCR experiments were used to prove the reliability of the network. For example, Chr4: 150439343‐150534945+, Chr18: 12871078‐12898301‐, Chr3: 59031546‐59042413+, Chr2: 140042094‐140057499‐ and Chr6: 119920110‐119921028+ were upregulated in RIP‐qPCR experiments. mRNAs (Lamp1, Cln3, Cln5, Litaf, Ctsb, Lipa) were also upregulated in RIP‐qPCR.

Heat shock factor, a regulator of heat shock response, maintains the stability of the intracellular environment by protecting cells from environmental stress or stress related to cell proliferation and differentiation.^53^ Heat shock transcription factor 4 (HSF4) is closely associated with lens development.^54^, ^55^ It controls the expression of heat shock proteins (Hsps), alpha β‐crystallin and γ‐crystallin in lens tissue.^55^ Mutations of HSF4 were associated with autosomal dominant cataracts.^56^ In lens, lysosomes are involved in maintaining the homeostasis of LEC and the terminal differentiation of fibre cell. HSF4 may participate in protein and nuclear DNA quality control by regulating the alpha β‐crystallin‐associated lysosomal pathway.^57^

In this study, HSF4 was decreased in the lens of *AQP5* ^−/−^ mice compared with that of *AQP5* ^+/+^ mice (Figure 7B). In addition, the expressions of Lamp1, Lipa, Gla and Cln5 were decreased in the lens and the primary lens epithelial of *AQP5* ^−/−^ mice (Figure 6D–F). Through the transcription factor prediction assay, we found that HSF4, as a transcription factor, regulated many mRNAs in lysosomal pathways (Figure 4). Finally, it was found that HSF4 was a target gene of miR‐149‐5p (Figure 7A–C). In bioinformatics analysis Chr16: 33421321‐33468183+ can inhibit miR‐149‐5p by sponge mechanism (Figure 7D,E). Chr16: 33421321‐33468183+ may regulate lysosome associated mRNAs by targeting the miR‐149‐5p/HSF4 axis (Figure 7F).

The present study discovered that deficiency of AQP5 causes early onset cataract in mice. *AQP5* knockout may be related to altering the level of autophagy in the lens, in addition circRNA levels were significantly changed in the lens of *AQP5* ^−/−^ mice. The data presented here indicate that *AQP5* may coordinate downstream regulatory events through circRNA‐miRNA‐mRNA network and HSF4‐mediated lysosome expression, which participated in the pathogenesis of abnormal lens development.

## AUTHOR CONTRIBUTIONS

**Hu Shaohua:** Methodology (equal); writing – original draft (equal); writing – review and editing (equal). **Wang Yihui:** Project administration (equal); writing – original draft (equal). **Zhang Kaier:** Investigation (equal); methodology (equal). **Bai Ying:** Methodology (equal). **Wang Xiaoyi:** Investigation (equal); validation (equal). **Zhao Hui:** Data curation (equal); supervision (equal). **Di Guohu:** Data curation (equal); writing – original draft (equal); writing – review and editing (equal). **Chen Peng:** Funding acquisition (lead); project administration (lead).

## CONFLICT OF INTEREST STATEMENT

The authors declared that they have no conflict of interest.

## Supporting information


**Figure S1.** Western blot bands for LC3II/I, P62 and GAPDH in the lenses of AQP5^+/+^ and AQP5^−/−^ mice.

**Figure S2.** Western blot bands for lysosomal proteins Lamp1, Cln5, Gla, Lipa and GAPDH in the lenses of AQP5^+/+^ and AQP5^−/−^ mice.

## Data Availability

All the raw data is available.
