# Supplementary figures and images for: Aquaporin 5 maintains lens transparency by regulating the lysosomal pathway using circRNA
Source: J Cell Mol Med. 2023 Feb 23;27(6):803–18. doi: 10.1111/jcmm.17679 (PMC10002928; doi:10.1111/jcmm.17679)

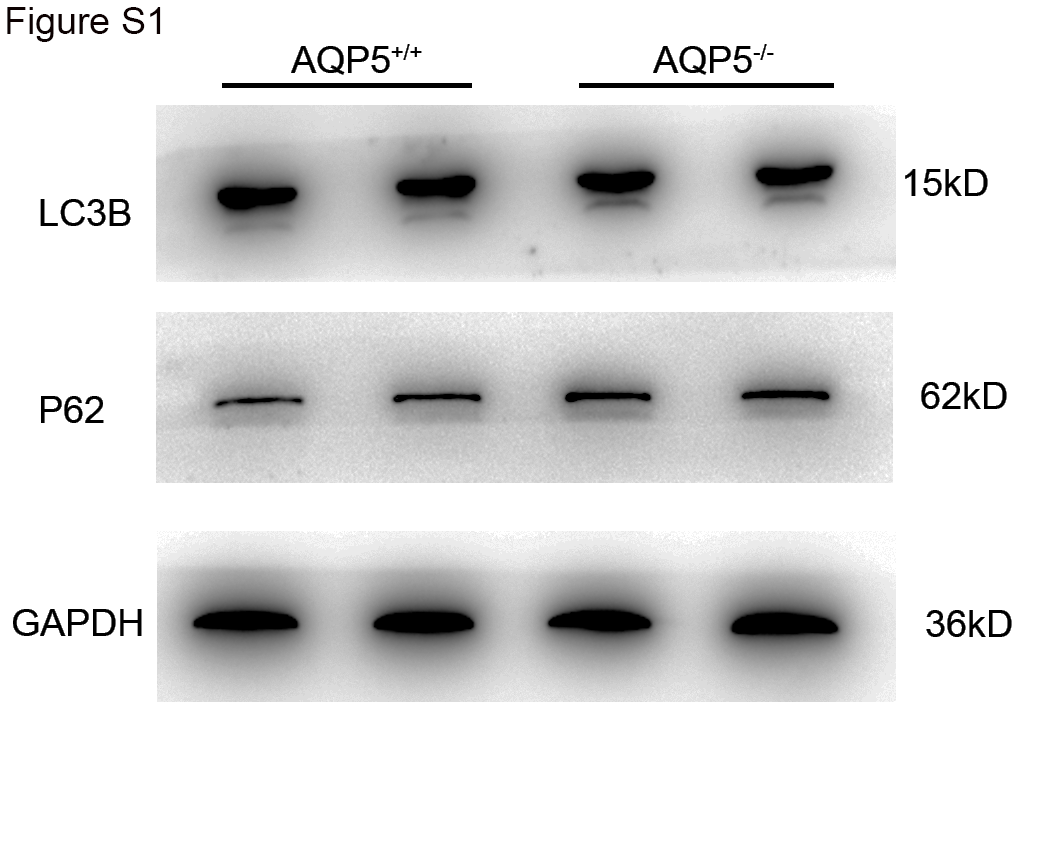

Supplement: Supplementary file 1 — Figure S1. Western blot bands for LC3II/I, P62 and GAPDH in the lenses of AQP5+/+ and AQP5−/− mice. [file JCMM-27-803-s002.tif]

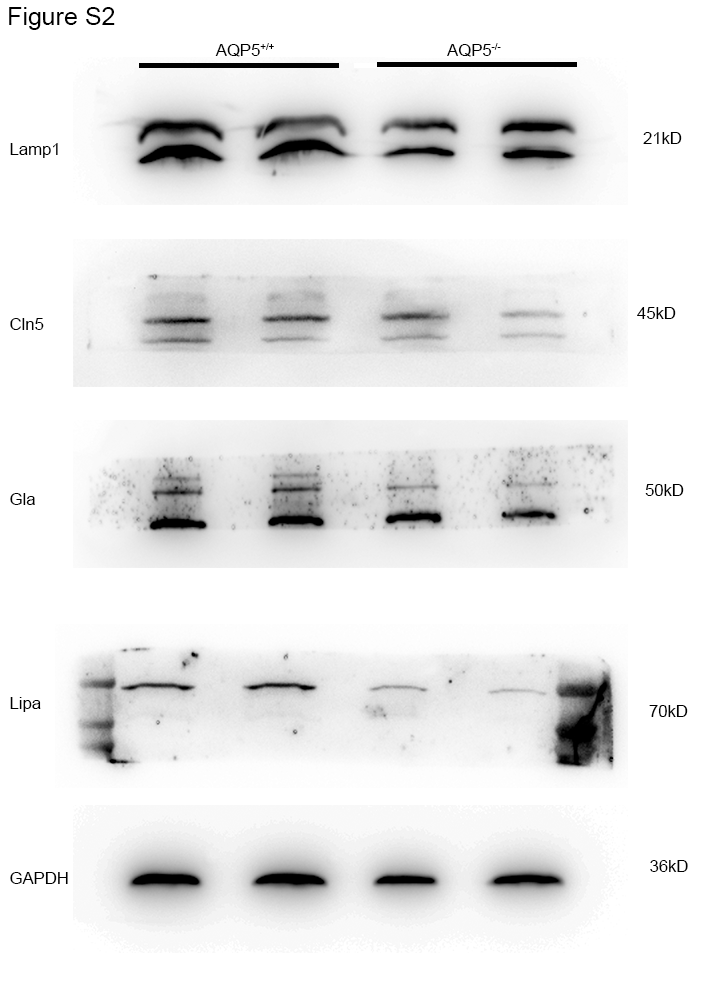

Supplement: Supplementary file 2 — Figure S2. Western blot bands for lysosomal proteins Lamp1, Cln5, Gla, Lipa and GAPDH in the lenses of AQP5+/+ and AQP5−/− mice. [file JCMM-27-803-s001.tif]
